# HTLV-1 associated acute adult T-cell lymphoma/leukemia presenting as acute liver failure in Micronesian: A case report

**DOI:** 10.1097/MD.0000000000026236

**Published:** 2021-07-16

**Authors:** Arash Ghaffari-Rafi, Young Soo Rho, Andrew Hall, Nicolas Villanueva, Masayuki Nogi

**Affiliations:** a University of California, Davis, School of Medicine, Department of Neurological Surgery Sacramento, CA; b University of Hawai’i at Mānoa, John A. Burns School of Medicine Honolulu, Hawaii; c University of Hawai’i at Mānoa, John A. Burns School of Medicine, Department of Medicine Honolulu, Hawaii; d The Queen's Medical Center Honolulu, Hawaii; e University of Hawai’i at Mānoa, John A. Burns School of Medicine, Department of Pathology Honolulu, Hawaii.

**Keywords:** acute hepatitis, acute liver failure, adult T-cell lymphoma/leukemia, Hawaii, human T-cell lymphocytic virus-1, hyperbilirubinemia, marshallese, micronesia

## Abstract

**Rationale:**

Malignant infiltration accounts for 0.5% of acute liver failure cases, with non-Hodgkin's lymphoma the predominant cause. Adult T-cell lymphoma/leukemia (ATLL) is a rarer source of acute hepatitis, with only 3 cases reported and all resulting in immediate deterioration with death. ATLL rises from human T-lymphocytic virus-1 (HTLV-1), commonly found in Japan (southern and northern islands), the Caribbean, Central and South America, intertropical Africa, Romania, and northern Iran. In Micronesia, HTLV-1 infection amongst native-born is absent or exceedingly rare.

**Patient Concerns:**

A 77-year-old Marshallese man presented to the emergency department with a 1-week history of generalized weakness, fatigue, and nausea. The physical exam revealed a cervical papulonodular exanthem and scleral icterus.

**Diagnosis:**

Laboratory studies were remarkable for aspartate-aminotransferase of 230 IU/L (reference range [RR]: 0–40), alanine-aminotransferase of 227 IU/L (RR: 0–41), alkaline phosphatase of 133 IU/L (RR: 35–129), and total bilirubin of 4.7 mg/dL (RR: 0–1.2), supporting acute liver injury. Platelet count was 11.6x10^4^/μL (RR: 15.1–42.4 × 10^4^), hemoglobin was 13.8 g/dL (RR: 13.7–17.5), and white blood cell count was 7570/μL (RR: 3800–10,800) with 81.8% neutrophils (RR: 34.0–72.0) and 10.4% lymphocytes (RR: 12.0–44.0). The peripheral blood smear demonstrated abnormal lymphocytes with occasional flower cell morphology. HTLV-1/2 antibody tested positive. The skin and liver biopsies confirmed atypical T-cell infiltrate. The diagnosis of ATLL was established.

**Interventions:**

The patient elected for palliative chemotherapy with cyclophosphamide, vincristine, and prednisone (CVP). He began antiviral treatment with zidovudine 250 mg bis in die (BID) indefinitely. Ursodiol and cholestyramine were added for his hyperbilirubinemia.

**Outcomes:**

Four weeks from admission, the patient returned to near baseline functional status and was discharged home.

**Lessons:**

This case highlights that ATLL can initially present as isolated acute hepatitis, and how careful examination of peripheral blood-smear may elucidate hepatitis etiology. We also present support for utilizing ursodiol with cholestyramine for treating a hyperbilirubinemia. Moreover, unlike prior reports of ATLL presenting as liver dysfunction, combined antiviral and CVP chemotherapy was effective in this case. Lastly, there are seldom demographic reports of HTLV-1 infection from the Micronesian area, and our case represents the first indexed case of HTLV-1-associated-ATLL presenting as acute liver failure in a Marshallese patient.

## 1 Introduction

The first human retrovirus to be identified, human T-lymphocytic virus (HTLV) was isolated in the United States (1980) and Japan (1982) independently.^[1–3]^ Soon after, HTLV type 1 (HTLV-1) was confirmed as the etiology behind the geographic clustering of a distinct leukemia in southwest Japan, termed adult T-cell leukemia/lymphoma (ATLL).^[4]^ Today, 5 to 10 million people worldwide are estimated to be infected with HTLV-1, with numbers relatively unknown in India, China, and several other densely populated regions.^[5]^ Despite the global distribution, HTLV-1 is found in minute foci of high infection prevalence surrounded by low endemic areas (i.e., Mashhad, Iran; Okinawa, Japan; Alice Springs, Australia; Tumaco, Colombia).^[6–9]^ This unusual geographic spread is theorized to arise from the founder effect.^[5]^

Irrespective of distribution, HTLV-1 infection is associated with numerous diseases, including: ATLL, HTLV-1-associated myelopathy/tropical spastic paraparesis, HTLV-1-associated uveitis, dermatologic conditions (i.e., infective dermatitis, crusted scabies), opportunistic infections, rheumatic/autoimmune conditions (i.e., Sjogren's syndrome, rheumatoid arthritis), inclusion body myositis and polymyositis, polyneuropathy, and depression.^[10–22]^ ATLL itself is further classified into 4 clinical variants (acute, lymphomatous, chronic, and smoldering), with acute being the most common.^[3,23]^ Typically, acute ATLL is characterized by leukocytosis, skin involvement, and generalized lymphadenomegaly; also often accompanied are hepatosplenomegaly, hypercalcemia, and elevated lactic dehydrogenase (LDH).^[3,24]^

This case report emphasizes that acute ATLL can present as isolated acute hepatitis without hepatosplenomegaly or an abnormal white blood cell count. Moreover, we highlight how meticulous peripheral blood-smear examination may decipher the cause behind hepatitis of unknown etiology. Unlike the 3 prior reports of ATLL presenting as liver dysfunction, combined antiviral with chemotherapy (cyclophosphamide, vincristine, and prednisone; [CVP]) was effective in our case.^[25–27]^ Furthermore, HTLV-1 infection in Micronesia is reported as absent or rare (with no data from the Marshall Islands), our case represents the second indexing of HTLV-1-associated-ATLL in a Marshallese patient.^[28–31]^ We hope to increase awareness that this life-threatening condition can not only present solely as acute hepatitis, but also in nontraditional populations (i.e., Marshallese and/or Micronesians).

## 2 Case report

We present a 77-year old man who was brought to the emergency department (ED) by his wife. He presented with the chief complaint of slowly progressive fatigue and generalized weakness for the past 7-days. At baseline the patient was fully independent without disability, but the worsening progression of symptoms caused the patient to become bedbound and was the impetus for going to the ED.

The patient denied any inciting events, including new medications or supplements (including kava), changes to diet, recent travel, sick contacts, or exposure to standing water. Associated symptoms included lightheadedness and mild nausea, but no vomiting.

His past medical history was notable for benign prostatic hyperplasia, hypothyroidism, right thyroid goiter, hypercholesterolemia, and impaired fasting glucose. Past surgeries included transurethral resection of the prostate and right thyroid biopsy. He denied prior traumas, transfusions, and allergies. Immunizations were up-to-date and he had regular follow-up with his primary care provider. His medications included levothyroxine 75 mcg daily, doxazosin 4 mg daily, finasteride 5 mg daily, and pravastatin 40 mg daily. His mother and father both passed away from unknown cancers, his brother has a gastrointestinal stromal tumor, and his adult children have no known health conditions.

The patient is of native Marshallese descent, born and raised in the Marshall Islands, and immigrated to Hawaii 50-years prior; his family denies any known Japanese ancestry. He lives at home with his wife. He was a former smoker, with 10 to 25 pack years, quitting 37-years ago. He denies any recent and former alcohol or drug use. He is independent with respect to his instrumental activities of daily living.

**Figure 1:**
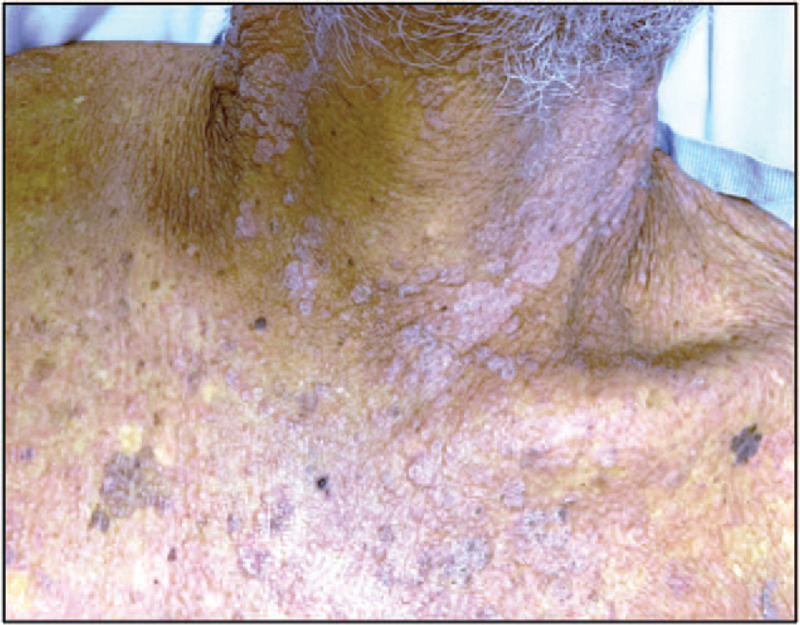
Papulonodular exanthem. Patient exhibited a new (appeared within past 1 month) upper trunk and proximal extremity papulonodular rash circumferentially encompassing the neck and C2-C4 dermatomes. After biopsy the rash was identified as leukemia cutis.

Patient was lethargic, weak-appearing, thin, and slow in answering questions, but not in acute distress. His sclera was icteric, while cardiac and lungs exams were unremarkable. Abdomen was soft, non-tender, non-distended, without a palpable liver or spleen. Peripheral pulses were 2+ (0–4 grading; 2+ indicates slightly more diminished then normal) with no extremity edema. Cervical, supraclavicular, axillary, and inguinal lymph nodes were not palpable. Skin was remarkable for a cervical papulonodular rash expanding circumferentially around the neck, in the C2-C4 dermatome distribution (Fig. 1).

On admission the patient's complete blood count was notable for a white blood cell count of 7570/μL (reference range [RR]: 3800–10800) with 81.8% neutrophils (RR: 34.0–72.0) and 10.4% lymphocytes (RR: 12.0–44.0). His hepatic function profile was remarkable for an aspartate-aminotransferase of 230 IU/L (RR: 0–40), alanine-aminotransferase of 227 IU/L (RR: 0–41), and a total-bilirubin of 4.7 mg/dL (RR: 0–1.2). His basic metabolic panel, magnesium, urinalysis, and influenza type A and B assessments were unremarkable. His transaminitis and hyperbilirubinemia were extensively worked-up.

**Figure 2:**
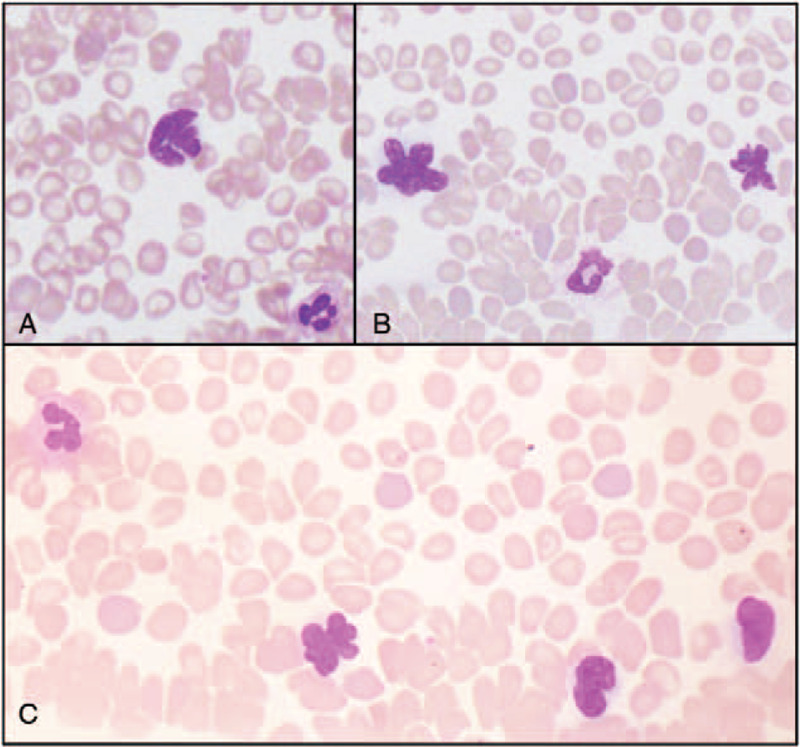
Peripheral blood smear. Abnormal lymphocytes with occasional flower cell morphology is observed in the panels A, B, and C.

A battery of tests were conducted in assessing for possible causes of hepatic insult and etiology of chief complaint, however results were inconclusive (Table 1). Subsequently, a peripheral blood smear was obtained, which exhibited occasional abnormal lymphocytes with flower cell morphology (Fig. 2). Immunophenotyping by flow cytometry was consistent with mature T-cell leukemia/lymphoma. Flow cytometric evaluation presented an abnormal CD45 bright lymphoid population (small-moderate in size by light scatter; 33% of all cells, 70% of lymphocytes) which expressed CD2, CD3, CD4, CD5, CD25, CD30 (weak), CD43, HLA-DR (weak), and alpha-beta T-cell receptor. There was no significant expression for CD1a, CD7, CD8, CD34, gamma-delta T-cell receptor or natural killer cell markers (CD16, CD56, and CD57). Also present were B-cells without demonstratable surface light chain restriction, along with a population of CD56+/CD3- natural killer cells (6% of lymphocytes). A distinct population of CD34 positive blasts (<0.1%) was not identified.

**Table 1:** Diagnostics. A series of tests were conducted to elucidate etiology of the patient's hepatic injury. However, the results were inconclusive.

Diagnostic tests	Results
Acetaminophen (Tylenol) level	<5 μg (RR: 10–30)
Plasma/serum ethanol	Negative
Gamma-glutamyl transferase	51 IU/L (RR: 8–61)
Comprehensive urine drug screen	Negative
Blood mercury	13 mcg/L (RR: < 11)
Serum copper	58 mcg/dL (RR: 70–175)
Ceruloplasmin	19 mg/dL (RR: 15–30)
Midnight cortisol	13.0 $\frac{\mu \text{g}}{\text{dL}}$ (RR: 3.0–16.0)
HIV-1/2 antigen/antibody	Negative
Vitamin B12	>2000 pg/mL (RR: 232–1245)
Serum folate	7.0 ng/mL (RR: > 3.1)
Haptoglobin	<10 mg/dL (RR: 30–200)
Epstein-Barr virus (EBV) quantitative DNA polymerase chain reaction (PCR)	<200 copies/mL (RR: <200)
Rapid plasma reagin	Non-reactive
Acute hepatitis panel	Negative: hepatitis A virus antibody IgM, hepatitis B core antibody IgM, hepatitis B surface antigen, hepatitis C antibody
Hepatitis E virus IgM	Not detected
Hepatitis E virus IgG	Not detected
Hepatitis D virus antibody	Negative
Throat herpes simplex virus (HSV) type 1 and 2 by real-time PCR	HSV type 1 detected, HSV type 2 not detected
Anti-nuclear antibody titer	<40, RR: ≤40
Actin (smooth muscle) antibody IgG	<20 U, RR: ≤20
Mitochondrial antibody	Negative
Liver kidney microsome antibody IgG	Negative at <20.0, RR: ≤20.0
IgM	25 mg/dL, RR: 40–230
IgG	773 mg/dL, 700–1600
IgA	169 mg/dL, RR: 70–400
Alpha-1 antitrypsin	87 mg/dL, RR: 90–200
Alpha-1 antitrypsin phenotype	PI∗MM (90% of normal individuals have the MM phenotype)
EBV antibody to early antigen IgG ratio	<0.2 (negative, RR: ≤0.8)
EBV antibody to nuclear antigen IgG ratio	>8.0 (positive, RR: ≤0.8; indicating past exposure)
EBV antibody to viral capsid antigen (VCA) IgG ratio	>8.0 (positive, RR ≤0.8; indicating immunological exposure either as silent primary infection or past exposure)
EBV antibody to VCA IgM ratio	<0.2 (negative, RR ≤0.8)
Transferrin	150 mg/dL (RR: 200–360)
Ferritin	1267 ng/mL (RR: 30–400)
Total iron	179 $\frac{\mu \text{g}}{\text{dL}}$ (RR: 45–160)
Iron binding capacity	<196 $\frac{\mu \text{g}}{\text{dL}}$ (RR: 228-428
Percent saturation (iron studies)	>91.3% (RR: 20–50)

**Figure 3:**
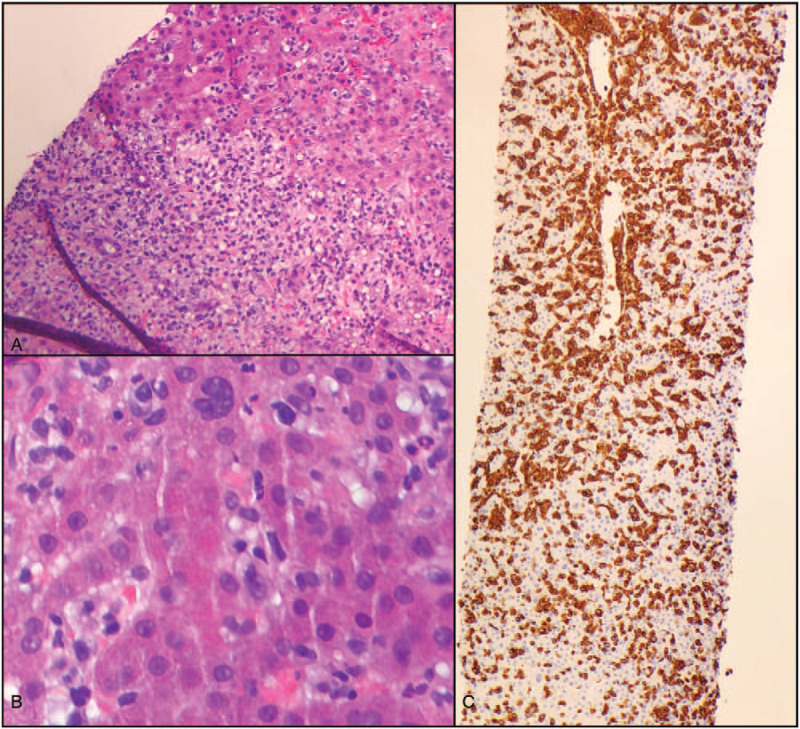
Liver biopsy. (A and B), demonstrate liver parenchyma with a sinusoidal and portal infiltrate composed of atypical lymphocytes along with scattered neutrophils. The atypical lymphocytes stained (not shown) positively for CD2, CD3, CD4, CD5, and CD30, with Ki67 significantly increased (> 90%). ALK1 was negative. C, CD3 (T-cell marker) highlights the atypical lymphocyte infiltrate.

**Figure 4:**
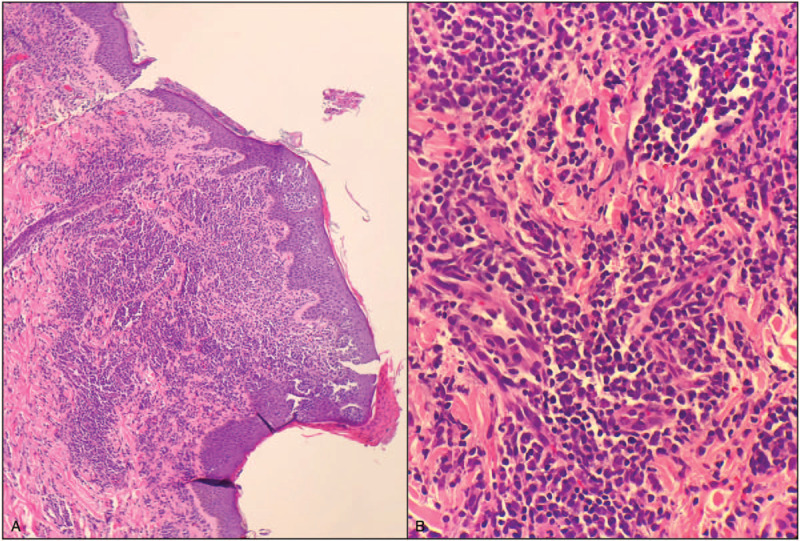
Skin biopsy. (A), low magnification shows an atypical cellular infiltrate in the epidermis and dermis. (B), high power demonstrates the infiltrate to be composed of medium sized, mildly pleomorphic lymphocytes. Immunohistochemical staining exhibited lymphocytes positive for CD3, CD4, CD8, and CD20, with occasional large cells positive for CD30, hence consistent with adult T-cell lymphoma/leukemia.

Following, a liver biopsy (Fig. 3) showed hepatic parenchyma with a sinusoidal and portal infiltrate of atypical lymphocytes, along with a number of neutrophils. The atypical lymphocytes were medium-large in size with significant nuclear pleomorphism. Immunohistochemistry showed the atypical lymphocytes to be positive for CD2, CD3, CD4, CD5, and CD30. Ki67 was significantly increased (>90%) in the atypical cells. PAX-5 and CD20 highlighted rare scatted B-cells, while CD7, CD8, CD56, and CD57 highlighted rare scattered natural killer cells. ALK1 was negative. Liver iron staining was graded as 2+ (mild; RR: 0 to 4+), while reticulin and trichrome stains demonstrating no obvious fibrosis. Meanwhile, skin biopsy (Fig. 4) of the left neck trapezius revealed an atypical T-cell infiltrate consistent with T-cell leukemia/lymphoma. Immunophenotypic studies highlighted a CD4 predominant T-cell infiltrate (CD3, CD4, CD8, CD20), with large cells positive for CD30.

**Figure 5:**
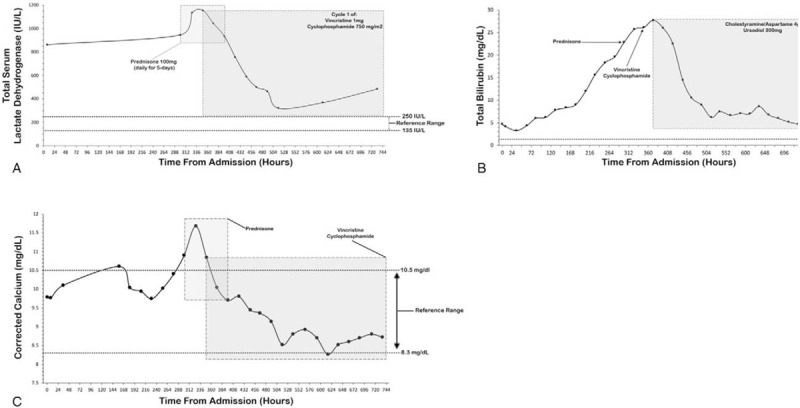
Trended Serum LDH, Total Bilirubin, and Corrected Calcium. (A), serum LDH down-trended upon initiation of the CVP chemotherapy regimen. (B), total bilirubin continued rising despite initiating chemotherapy, but began down-trending upon initiation of ursodiol and cholestyramine/aspartame. (C), corrected calcium was not elevated on admission, but spiked briefly with the initiation of prednisone, likely secondary to tumor lysis.

HTLV I/II antibody Western blot resulted positive for HTLV-1 antibodies, consistent with HTLV-1 infection, with the patient being considered infectious. The patient was started on palliative chemotherapy, involving a 21-day cycle of prednisone 100 mg (for 5 days), vincristine 1 mg (dose reduced for hyperbilirubinemia), and cyclophosphamide 750 $\frac{\text{m}{\text{g}}^{2}}{\text{m}}$. Due to the patient's deteriorating condition, prednisone was initiated prior to confirmation of ATLL, yet there was no improvement in total bilirubin; upon ATLL confirmation, he was then started on cyclophosphamide and vincristine for cytoreduction. Doxorubicin was held due to hyperbilirubinemia. Zidovudine 250 mg bis in die (BID) was initiated (upon return of HTLV-1 results) to reduce HTLV-1 burden. With initiation of the CVP regimen the patient's total lactate dehydrogenase (LDH) and corrected calcium (Fig. 5A, 5C) declined. However, total bilirubin continued rising to a peak of 27.7 mg/dL and eventually declined after the addition of oral cholestyramine/aspartame 4 g daily and ursodiol 300 mg daily (Fig. 5B). Asterixis and hepatic encephalopathy were managed with lactulose 20 g ter in die and rifaximin 550 mg BID.

The treatment course was complicated by severe anemia that was out of proportion of chemotherapy induced pancytopenia. Esophagogastroduodenoscopy demonstrated esophagitis, gastritis, and non-bleeding gastric and duodenal ulcers as the source of hemorrhage. Oral mucosa revealed extensive candida. Stool ova and parasite test were negative for *Strongyloides stercoralis* infection. Tissue biopsy from oropharyngeal area confirmed involvement of herpes simplex virus and Candida which were treated with acyclovir and anidulafungin. Chemotherapy induced bone marrow suppression was treated with filgrastim-sndz. The patient returned to near baseline and was discharge after 4-weeks from hospitalization. However, within 4-weeks of discharge the patient experienced a cardiac arrest at home and passed away.

## 3 Discussion

### 3.1 Epidemiology

Distribution of ATLL parallels the prevalence of HTLV-1 infection.^[3]^ Major endemic regions of HTLV-1 include west and central Africa (i.e., Gabon [25% in some Haut-Ogoué region villages], Democratic Republic of the Congo [0.7%–3.7%], Nigeria [5.5%], Ghana [1%–2.7%], Senegal [0.2%–1.2%], Guinea [0.2%–1.9%], Ivory Coast [1%–2.7%], and Cameroon [0.5%–2%]), South America (i.e., Brazil [1.8% in Salvador, Bahia], Peru [1.3%–3.8%], Chile [0.7%–1.9%]), the Caribbean (i.e., Jamaica [6.1%], Barbados [2%], Martinique [2%], Guadeloupe [2%], Trinidad [2%], and Tobago [2%]), and southern Japan [1%–6%].^[5,32,33]^ Meanwhile, in several nations isolated pockets of endemicity can be found surrounded by near absence of HTLV-1 infection: Urmia, Azerbaijan (Iran) [0.34%]; Mashhad, Khorasan (Iran) [1.97%]; Alice Springs, Australia [0.32%]; Fujian, China [0.001%]; Tumaco, Colombia [2.8%].^[7,9,34–36]^ Although few longitudinal studies have been conducted, one 5-year study identified HTLV-1 seroprevalence to be decreasing in Iran, even in endemic centers.^[34]^

Moreover, within Europe and North America HTLV-1 infection is found primarily amongst individuals with heritage from endemic regions. In Europe, excluding Romania—the only endemic nation [0.00053%]—studies from the United Kingdom, France, and Spain identified over 80% of infections to originate from citizens with heritage from endemic locations—primarily the West Indies (i.e., Jamaica, Barbados, Martinique, Guadeloupe, Trinidad, and Tobago), Africa (i.e., Ghana, Sierra Leone, Senegal, Mali, Guinea, Ivory Coast, and Cameroon), and South America.^[5,37,38]^ Additionally, another 5% to 10% of infections were accounted for by Caucasian women who acquired HTLV-1 through sexual transmission from a partner who originated from an endemic region.^[39]^ Similarly, in mainland United States, HTLV-1 cases are found predominantly in Florida and New York, with those infected usually having Afro-Caribbean or African-American heritage.^[5,40]^

In Hawaii, HTLV-1 infection is found amongst patients of southern Japanese heritage.^[41–44]^ Our case represents the second indexing of ATLL in a Micronesian (i.e., Marshallese).^[28]^ Although our patient denies knowledge of having Japanese ancestry, at the outbreak of World War I (1914) and through World War II (1945), Japan occupied the Marshall Islands, resulting in 5.9% of today's Marshallese having mixed ancestry.^[45,46]^ Currently, studies indicate HTLV-1 infection amongst native Micronesians and Polynesians to be absent or incredibly rare.^[29]^ By genotyping the virus, the origin of our patient's infection may have been imputed via molecular epidemiology.

Four geographically distinct genotypes of HTLV-1 exist: Cosmopolitan subtype A (divided into the Transcontinental, Japanese, West African, and North African subgroups), Central African subtype B, Central African/Pygmies subtype D, and Australo-Melanesian subtype C.^[5]^ If our patient acquired HTLV-1 through Japanese heritage his virus would likely be part of the Japanese or Transcontinental subgroups, genotypes both found in Japan.^[5]^ If he was fully native Marshallese, he may have carried subtype C, which would indicate the migration of the virus across the Pacific from Melanesia to Micronesia.

### 3.2 Transmission

Vertical transmission via breastfeeding constitutes the most common route for HTLV-1 infection, with intrauterine or peripartum transmission exceedingly rare (less than 5% of cases).^[47]^ Duration of breastfeeding (≥ 12 months, 24%–32% transmission rate; < 12 months, 5%–9%), mother's proviral load, and HLA class I type mother-child concordance, all modify transmission efficacy.^[48–51]^ Other sources of transmission include blood contact via transfusions or sharing of needles/syringes.^[52]^ Although efficacy of transmission via needle sharing in intravenous drug users is low, transfusion of infected blood produces yields infection in 50% to 60% of recipients.^[53,54]^ As viable lymphocytes are required for HTLV-1 infection, efficacy of transmission drops after storing blood products for more than 1 week.^[55]^ Lastly, sexual intercourse also provides an important transmission route, as HTLV-1 has been found in both cervical secretions and semen, with male-to-female transmission more efficacious than female-to-male.^[56]^

Notably, HTLV-1 infection route is associated with specific diseases. HTLV-1-associated myelopathy/tropical spastic paraparesis is linked to blood transfusions, while ATLL is associated with breastfeeding, with post-transfusion ATLL unprecedented.^[47,57,58]^ Amongst HTLV-1 carriers, most remain asymptomatic, with lifetime risk of 3% to 5% for developing ATLL.^[59]^ With a diagnosis of ATLL, along with our patient's history precluding transfusions or intravenous drug use, his HTLV-1 was likely acquired via breastfeeding.

### 3.3 Clinical presentation of adult T-cell lymphoma/leukemia

Due to the diversity in presentation and prognosis, ATLL has been categorized into 4 subtypes per the Shimoyama classification system: smoldering, chronic (leukemic), acute (leukemic), and lymphomatous.^[23]^ The smoldering subtype is characterized by presence of ≥5% abnormal lymphocytes on peripheral blood smear (or <5% if skin/lung infiltration is involved), a normal leukocyte count, a LDH value ≤1.5 times the upper limit of normal (ULN), no lymphadenopathy, no liver, spleen, central nervous system (CNS), bone, or gastrointestinal involvement, and no ascites or pleural effusion.^[23]^ Meanwhile, chronic ATLL is defined by leukocytosis, absolute lymphocytosis, a LDH ≥2 times the ULN, no hypercalcemia, no bone, gastrointestinal, or CNS involvement, and no ascites or pleural effusion; lymphadenopathy, liver, spleen, and skin involvement, along with ≥5% abnormal lymphocytes on peripheral blood smear may be present.^[23]^ Both chronic and smoldering types exhibit favorable prognoses, with 2- and 4-year survival rates of 52.4% and 26.9% in chronic, and 77.7% and 62.8% in smoldering.^[23]^ The presence of ascites and pleural effusion precluded the diagnosis of smoldering or chronic ATLL in our patient.

Lymphomatous and acute subtypes are considered the aggressive forms of ATLL, with respective 2- and 4-year survival rates of 21.3% and 5.7% for lymphomatous, and 16.7% and 5.0% for acute.^[23,24]^ The lymphoma subtype is characterized by no lymphocytosis, ≤1% abnormal lymphocytes, and massive lymphadenopathy (>1.5 cm).^[23]^ Our patient lacked lymphadenopathy, including on computed tomography imaging of his head, neck, chest, and abdomen. Hence, our patient was left with the diagnosis of acute ATLL, the most common type, accounting for 60% to 65% of cases.^[24,60]^ Acute ATLL is reserved for patients who do not meet criteria for the other 3 classifications.^[23]^

Acute ATLL is generally characterized by extensive lymphadenopathy, hepatosplenomegaly, leukocytosis, systemic symptoms (i.e., fever, night sweats, weight loss, and weakness), lytic bone lesions, and diffuse visceral involvement (skin, gastrointestinal tract, and lung infiltration).^[23,24,60]^ Clinical manifestations of severe hypercalcemia (i.e., renal dysfunction, neuropsychiatric alterations) are present in half of patients and generally the presenting symptoms.^[24,60]^

Our case was highly unusual in that the patient presented only with acute hepatitis and a new papulonodular rash; he had a normal lymphocyte count, no hepatosplenomegaly, no lymphadenopathy, no electrolyte abnormalities (including calcium), and no leukemic systemic symptoms (other than weakness). In the English literature, only 3 other cases of ATLL presenting with acute hepatitis have been indexed, yet in these cases other symptoms including lymphadenopathy, hepatosplenomegaly, fever, rigors, or hypercalcemia were also present.^[25,26,61]^ Hence, this report is the first to describe ATLL presenting exclusively as acute hepatitis with a papulonodular rash (Fig. 1), as well as the first to describe ATLL presenting with acute hepatitis in a Marshallese patient.

Malignant infiltration accounts for only 0.5% of acute liver failure cases, with most cases secondary to non-Hodgkin's lymphoma.^[62]^ Thus, ATLL induced acute liver failure is exceedingly rare. However, in certain regions of Japan where HTLV-1 is highly endemic, the incidence of ATLL infiltrating the liver is greater than non-Hodgkin's lymphoma.^[63]^ Hepatically, ATLL cells generally localize to the periportal region, resulting in massive hepatic ischemia and eventual widespread necrosis of the hepatocytes (suspected secondary to the Schwartzman phenomena).^[64]^ ATLL (versus non-Hodgkin's lymphoma) liver infiltration portends a poorer prognosis, possibly secondary to the risk of sepsis-induced hepatopathy, disseminated intravascular coagulation, or hemophagocytosis syndrome—complications not present in our patient.^[63,65–67]^

Overall, our case highlights how in situations where etiology of liver failure remains enigmatic, the clinical team should consider a peripheral blood smear to probe possibility of a hematologic malignancy (Fig. 2).

### 3.4 Diagnosis

At the 13th International Conference on Human Retrovirology: HTLV (2008), ATLL researchers drafted a consensus statement on management of the disease.^[16]^ To diagnosis ATLL (excluding lymphomatous subtype), ATLL-cells (extensively polylobulated nuclei containing condensed homogenous chromatin, with absent/small nucleoli and agranular/basophilic cytoplasm) must be detected in the peripheral blood; notably, flower cells are pathognomonic (Fig. 2).^[16]^ To secure the clinical diagnosis of ATLL, the patient must be seropositive for HTLV-1 with histologically/cytologically confirmed peripheral T-cell malignancy.^[16]^ The minimum requirements for ATLL diagnosis via immunophenotyping involves analysis of CD3, CD4, CD7, CD8, and CD25.^[16]^ ATLL cells are a mature CD4 T-cell population exhibiting CD2, CD5, CD25, CD45RO, CD29, T-cell receptor, and HLA-DR.^[16]^ In accordance, our patient's cells expressed CD2, CD3, CD4, CD5, CD25, CD30 (weakly), CD43, HLA-DR (weakly), and alpha-beta T-cell receptor. Lastly, although not required due to accessibility, cytogenetic analysis and testing for monoclonal integration of HTLV-1 proviral DNA are recommended when possible.^[16]^ Unfortunately, testing for monoclonal integration, cytogenetic analysis, and HTLV-1 genotyping were all unavailable at our facility. Regardless, our patient did meet diagnostic criteria for acute ATLL, and thus began treatment.

### 3.5 Treatment

Treatment regimens for acute ATLL must be determined on a case-by-case basis, taking into account prognostic factors (i.e., age, performance status, serum calcium, and LDH), exclusion criteria nuances from prior clinical trials, and contraindications to chemotherapeutics.^[3,16,24]^ As the best treatment regimen is unknown, patients are encouraged to enroll in clinical trials when possible.^[16]^ Regarding survival outcomes, LSG-15 (VCAP-AMP-VECP) presents the longest survival time (10.9 months) in acute ATLL patients, yet because patients with renal dysfunction were excluded and several of the medications utilized are not available outside of Japan (including the United States), treatment standard of care is not yet established; recent unpublished data indicates etoposide, prednisone, vincristine (Oncovin), cyclophosphamide, doxorubicin hydrochloride (EPOCH) yields similar results.^[24,68,69]^ In our case, treatment options were limited by the patient's liver dysfunction, for etoposide and doxorubicin (in EPOCH) are unsafe with hyperbilirubinemia, hence dictating the rational for CVP selection: a 21-day cycle regimen involving prednisone 100 mg (for 5 days), cyclophosphamide 750 $\frac{\text{m}{\text{g}}^{2}}{\text{m}}$, and vincristine 1 mg. Moreover, due to meta-analysis data indicating enhanced treatment response and prolonged survival, zidovudine 250 mg BID was initiated indefinitely; although standard of care is unclear, some recommend an antiviral with interferon prior to starting chemotherapy, but our patient's precipitous decline in health warranted immediate initiation of chemotherapy, reduction of zidovudine dosage (900 mg/day recommended), and exclusion of interferon due to side effect profile.^[24]^ With chemotherapy initiation, LDH levels precipitously dropped from 1153 IU/L to 318 IU/L within 165 hours (roughly 7 days) (Fig. 1A). Likewise, serum calcium levels returned within normal limits after 72 hours of starting chemotherapy (Fig. 1C). CNS prophylaxis was also planned outpatient, for when acute ATLL relapses at a new site the CNS is involved more than half the time.^[24]^

### 3.6 Hyperbilirubinemia management

Although LDH and calcium levels responded to chemotherapy, total bilirubin continued to rise (Fig. 1B). The patient's hyperbilirubinemia worsened from admission, peaking at 27.7 mg/dL with diffuse pruritus and mucosal jaundice developing. The origin of the hyperbilirubinemia was likely secondary to malignant infiltration and hepatocellular injury, as aspartate-aminotransferase, alanine-aminotransferase, and international normalized ratio were elevated, and biliary tree obstruction was ruled out. Moreover, on admission the patient had proper renal function, which deteriorated only throughout the hospital course, indicating a possible etiology of bile cast nephropathy and/or hepatorenal syndrome. As the hyperbilirubinemia precluded administration of certain chemotherapeutics (i.e., doxorubicin, etoposide, as part of EPOCH), daily cholestyramine 4 g with ursodiol 300 mg was initiated. Immediately, decline in total bilirubin was observed, with values dropping to 4.7 mg/dL at discharge; however, the decline in bilirubin was only correlative, instead likely resulting from delayed response to chemotherapy and reduced hepatic tumor burden.

### 3.7 Opportunistic infections

As patient with ATLL are functionally immunocompromised, often patients succumb to opportunistic infections, including malignant strongyloidiasis, *Pneumocystis jiroveci* pneumonia, herpes, cytomegalovirus, fungi (i.e., Candida, disseminated cryptococcosis), toxoplasmosis, and bacterial infections (i.e., abscesses, sepsis).^[24,60]^ In particular, patients are recommended to have screening for *Strongyloides stercoralis* via a stool ova and parasite, and if positive treated with thiobendazole, ivermectin, or albendazole.^[24,60]^ Although our patient tested negative for *S. stercoralis* and had no clinically evident *P. jiroveci* pneumonia, he did develop herpes simplex virus esophagitis and oroesophageal candidiasis, which required treatment with acyclovir and anidulafungin. Overall, Japanese trials have recommended patients be on trimethoprim-sulfamethoxazole, valacyclovir, and antifungals to provide prophylaxis against *P. jiroveci* pneumonia, viral, and fungal infections.^[69]^

## 4 Conclusion

Overall, our case highlights how clinicians should remain vigilant that ATLL can not only present as isolated acute liver failure, but also in nontraditional populations. Unlike the 3 prior reports of ATLL presenting as liver dysfunction, combined antiviral with CVP chemotherapy was effective in our case.^[25–27]^ Furthermore, our case represents the second indexing of ATLL in a Marshallese patient, despite HTLV-1 infection exceedingly rare in Micronesia.^[28–31]^ Although only correlative, our experience indicates possible utility of ursodiol and cholestyramine as adjuncts to chemotherapy for intractable hyperbilirubinemia. Overall, we hope to increase awareness of the atypical presentations ATLL may assume.

## Author contributions

**Conceptualization:** Arash Ghaffari-Rafi, Young Soo Rho, Masayuki Nogi.

**Data curation:** Arash Ghaffari-Rafi, Young Soo Rho, Andrew Hall, Masayuki Nogi.

**Formal analysis:** Arash Ghaffari-Rafi, Andrew Hall, Nicolas Villanueva, Masayuki Nogi.

**Funding acquisition:** Arash Ghaffari-Rafi.

**Investigation:** Arash Ghaffari-Rafi, Young Soo Rho, Masayuki Nogi.

**Methodology:** Arash Ghaffari-Rafi, Young Soo Rho, Masayuki Nogi.

**Project administration:** Arash Ghaffari-Rafi, Masayuki Nogi.

**Resources:** Arash Ghaffari-Rafi, Young Soo Rho, Andrew Hall, Masayuki Nogi.

**Software:** Arash Ghaffari-Rafi.

**Supervision:** Arash Ghaffari-Rafi, Young Soo Rho, Masayuki Nogi.

**Validation:** Arash Ghaffari-Rafi, Masayuki Nogi.

**Visualization:** Arash Ghaffari-Rafi, Masayuki Nogi.

**Writing – original draft:** Arash Ghaffari-Rafi.

**Writing – review & editing:** Arash Ghaffari-Rafi, Young Soo Rho, Andrew Hall, Nicolas Villanueva, Masayuki Nogi.
